# Therapeutic management of the odontogenic keratocyst. An energetic approach with a conservative perspective and review of the current therapeutic options

**DOI:** 10.4317/jced.56722

**Published:** 2020-08-01

**Authors:** Jordi Borrás-Ferreres, Alba Sánchez-Torres, Javier Alberdi-Navarro, José-Manuel Aguirre-Urizar, Adalberto Mosqueda-Taylor, Cosme Gay-Escoda

**Affiliations:** 1 DDS. MS. Master’s Degree Program in Oral Surgery and Orofacial Implantology (EFHRE International University/FUCSO); 2 DDS, MS. Master of Oral Surgery and Orofacial Implantology. Associate Professor of the Oral Surgery Department, School of Dentistry, University of Barcelona, Spain; 3 DDS, MS, PhD, Assistant Professor of the Oral Medicine and Oral and Maxillofacial Pathology Unit, Dental Clinic Service, Department of Stomatology II, University of the Basque Country (UPV/EHU), Leioa, Spain; 4 MD, DDS, PhD. Chairman and Professor of the Oral Medicine and Oral and Maxillofacial Pathology Unit, Dental Clinic Service, Department of Stomatology II, University of the Basque Country (UPV/EHU), Leioa, Spain; 5 MD, DDS, MSc. Health Care Department, Universidad Autónoma Metropolitana Xochimilco, Mexico City, Mexico; 6 MD, DDS, MS, PhD, EBOS, OMFS. Chairman and Professor of the Oral and Maxillofacial Surgery Department, School of Dentistry, University of Barcelona. Director of Master’s Degree Program in Oral Surgery and Implantology (EFHRE International University/ FUCSO). Coordinator/Researcher of the IDIBELL Institute. Head of Oral and Maxillofacial Surgery and Implantology Department of the Teknon Medical Centre, Barcelona, Spain

## Abstract

**Background:**

Odontogenic keratocysts (OKC) are cystic lesions appearing in the jaws, usually asymptomatic with a progressive growth into the bone. Many of them are diagnosed by a routine radiological examination.

**Material and Methods:**

This study reports a 12-year-old girl that presented an asymptomatic large radiolucent unilocular lesion associated to the crown of 3.8 that caused displacement of the molar and the inferior alveolar canal. Differential diagnosis included OKC, unicystic ameloblastoma, ameloblastic fibroma, dentigerous cyst and orthokeratinized odontogenic cyst. Two surgical interventions were performed; first, a marsupialization, and 10 months after, the third molar extraction plus cyst enucleation, mucosa excision and the application of Carnoy’s solution.

**Results:**

The anatomopathological exam confirmed diagnosis of OKC. There was no evidence of recurrence after 2 years of follow-up.

**Conclusions:**

Marsupialization followed by surgical enucleation with mucosa excision and Carnoy’s solution can help manage treatment of OKC, a lesion characterized by an aggressive behavior.

** Key words:**Odontogenic keratocyst, mucosa excision, carnoy solution, third molar, tooth extraction.

## Introduction

Odontogenic keratocysts (OKC) are cystic lesions appearing in the jaws, classified as developmental cysts arising from the dental lamina (1). They represent approximately 11% of all odontogenic cysts and the age of onset has two peaks of maximum frequency, one between the second and third decades of life and other, less intense, around the fifth decade (2). These cysts are more frequent in men, with a ratio of 1:1.4, and they are more common in the mandible, with a predilection for the angle and ramus (3). This lesion may occur in association with an impacted third molar (4-6).

Radiographically, OKC appear as uni or multilocular radiolucent lesions that can adopt a wide range of morphologies, so radiological exam is not considered a pathognomonic test for the definitive diagnosis of OKC since several lesions may have similar characteristics (4,7,8). Generally, these lesions are asymptomatic and have a progressive growth into the bone, narrowing or bulging the bone corticals and, ocasionally, provoking bony erosion or resorption (9). Many of them are diagnosed by a routine radiological examination, although some, specially if very large or inflammed can cause swelling and pain (3,5,6,8). Histologically, OKC are characterized by a very thin fibrous capsule whose epithelial lining has a parakeratinized corrugated surface and a basal layer of cylindrical cells in palisade that demonstrate inverted polarity, at least focally (3).

Compared to other odontogenic cysts, OKC have an infiltrating growth pattern, an aggressive biological behavior and a greater tendency to recur after its elimination, which can reach up to 56% of cases after a simple enucleation (10). The delicate and fragile nature of its epithelial lining, weakly connected to the capsular connective tissue, makes these lesions prone to tearing, separating and breaking during surgical excision, sparing some epithelial remains which can lead to recurrence since they have a high proliferative activity (11). Recurrence may also be due to the persistence of satellite cysts that would remain during surgery, the presence of cystic debris in the adjacent bone or mucosa, or the existence of epithelial islands in the lining mucosa (2,8,9,11). Moreover, the fact that OKC arise from the inactivation of the chromosome 9q patched gene (PTCH), a tumor suppressor gene, led in 2005 to be classified as a keratocystic odontogenic tumor (12). However, this denomination has never reached great acceptance and currently, OKC has again considered a type of developing cyst instead an unequivocal neoplasm (1).

There are different surgical approaches to treat OKC. One of the conservative surgical treatments consists in the enucleation with or without the use of a complementary procedure, such as the application of Carnoy’s solution or liquid nitrogen (4,9,13-15). Marsupialization is another conservative modality, as well as decompression followed by enucleation (3,7,16,17). Although adjuvants to simple enucleation decrease the recurrence rate, they still exceed 10% (11). Occasionally, in extensive or recurrent cases, an aggressive surgical treatment based on a radical “in block” resection with reconstruction with a free or vascularized bone graft (5) would be indicated. Although amputation is the treatment modality with the lowest recurrence rate, between 0% and 8.4% (10,11), the subsequent reconstruction can produce important aesthetic and functional sequelae which can significantly alter the patient’s quality of life (5).

The main objective of this study was to show the clinical, radiographic and histological evolution of a pediatric patient with a large OKC treated by a conservative surgical therapy that combined distinct therapeutic variants to minimize the risk of recurrence.

## Case Report

**Figure 1:**
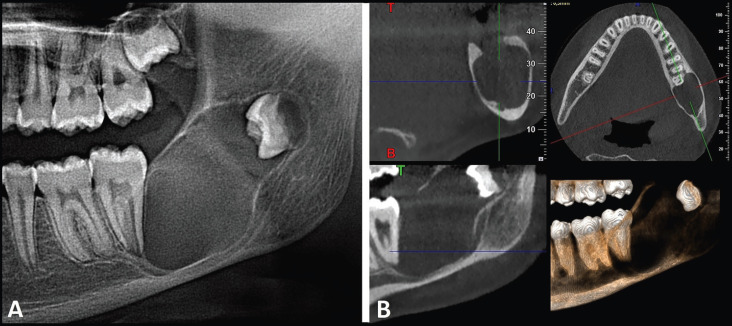
Radiological characteristics. A. Panoramic radiograph. B. Slides from cone-beam computed tomography.

A 12-year-old girl came to the dental clinic for a complete dental check-up. No oral pathology was observed at clinical examination. However, the panoramic radiograph disclosed a radiolucent, unilocular lesion with well-defined and corticated borders in the left mandibular angle. The lesion measured 3x2x1.5 cm in the anteroposterior, coronoapical and mediolateral planes respectively, associated to the crown of 3.8, which was displaced towards the ascending ramus. Besides, the lesion displaced the inferior alveolar canal and reached the roots of molar 3.7 (Fig. 1A). A cone-beam computed tomography showed that the lesion had reabsorbed part of the alveolar ridge, involving internal and external bone plates and expanding them (Fig. 1B). The differential diagnosis of the radiological image included OKC, unicystic ameloblastoma, ameloblastic fibroma, dentigerous cyst and orthokeratinized odontogenic cyst.

**Figure 2:**
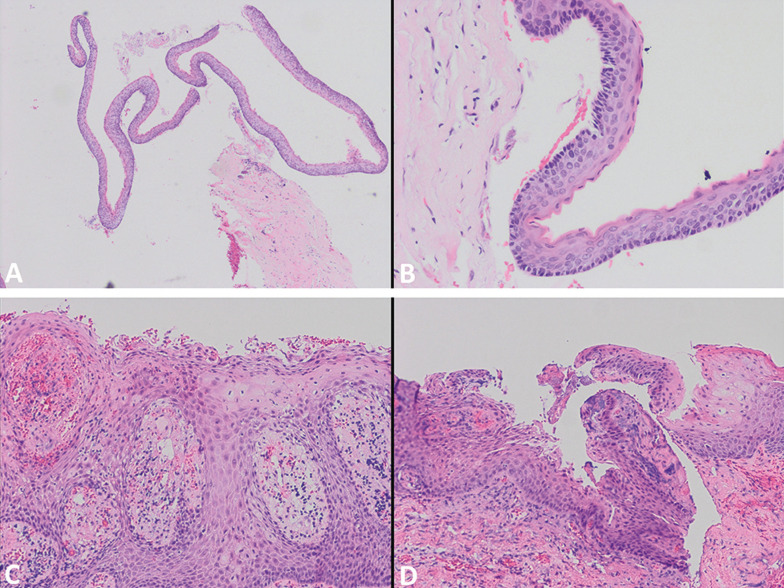
Anatomopathological exam of the incisional biopsy (A,B) and enucleation (C,D). A. Squamous epithelium detached from capsular connective tissue (H&E 20x). B. Squamous epithelium with superficial parakeratosis of corrugated aspect and palisade of basal cells. (H&E 40x). C. Squamous epithelium with loss of keratinization and lengthening of epithelial ridges. In the capsular connective tissue, a chronic inflammatory lymphoplasmocitary infiltrate is recognized. (H&E 40x). D. Squamous epithelium with focal parakeratosis, with areas of focal basal palisade. (H&E 40x).

**Figure 3:**
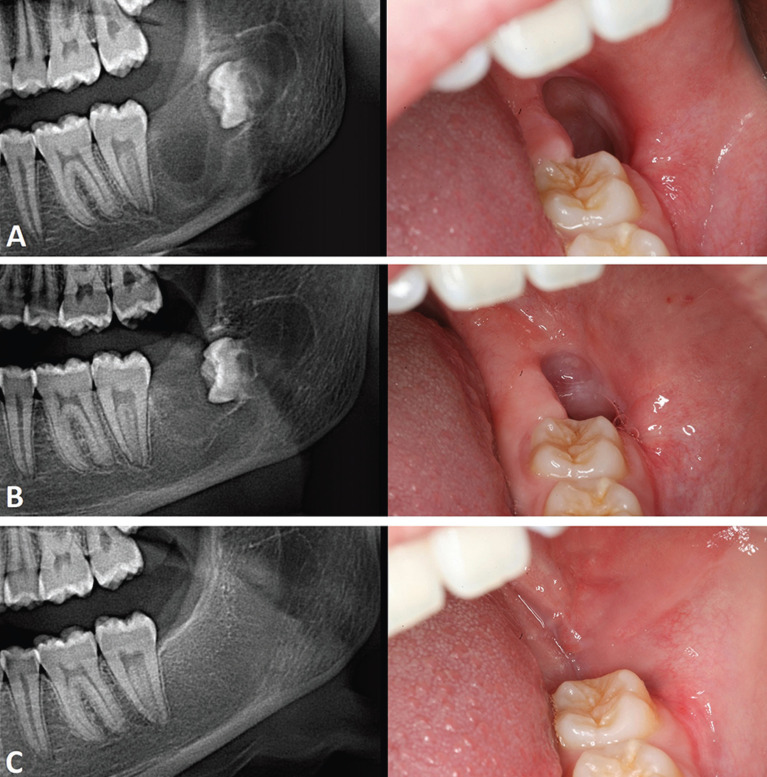
Clinical and radiological follow-up. A. Three months after marsupialization. B. Six months after marsupialization. C. Six months after the cyst enucleation.

Given the significant size of the lesion, a marsupialization with an incisional biopsy was performed. The surgical intervention was done under local anesthesia (4% articaine with epinephrine 1:200,000), using inferior alveolar and lingual nerve direct block technique. Subsequently, the buccal nerve was anesthetized by an infiltration on the vestibular mucosa of the retromolar trigone. The marsupialization consisted in a gingivectomy of the tissue covering the bone fenestration caused by the lesion. After the tissue excision, a large cavity lined by a thin capsule was evidenced. A yellowish-white material that was herniated, appeared inside the cavity. A sample of the capsule was taken and sent for its anatomopathological exam. Subsequently, the defect was filled with a sterile gauze embedded in chlorhexidine to prevent the oral mucosa from covering the lesion window. The characteristics of the capsule and the material inside constituted suggestive features of an OKC. The pieces analyzed corresponded to a fibrocellular connective wall lined by a well-defined polystratified epithelium with focally corrugated superficial parakeratosis, showing the basal cells in palisade. Focally, this epithelium appeared detached from the connective tissue showing several fragments of epithelium alone (Fig. 2A,B). Thus, the final diagnosis was OKC. Clinical and radiographic controls were performed every 3 months. The patient care throughout the process consisted in irrigating the cavity with a syringe filled with salty water 3 times a day and keeping it covered with a sterile gauze embedded in 0.12% chlorhexidine digluconate. During follow-up visits, the reduction of the lesion size was observed clinically and radiographically, with the formation of bone in its periphery and displacement of tooth 4.8 towards the alveolar crest (Fig. 3A,B).

Ten months after the onset of marsupialization, a complete cystectomy of the residual lesion was performed. At that time, the bone already covered the lower dental canal, a situation that prevented possible nerve damage during enucleation. The surgical intervention was performed under local anesthesia using the same technique. Since the initially performed window now contained the epithelial transition between the cystic and oral cavity, an elliptical incision was made 5 mm away from its edges, completely surrounding it until the healthy bone. This maneuver aimed to eliminate possible cystic and epithelial remnants located in the adjacent mucosa. Subsequently, the cyst was removed from the edges of the window, obtaining a complete enucleation of the lesion and eliminating the included third molar. Then, a chemical curettage of the bone cavity was carried out with Carnoy’s solution for 5 minutes, with the aim of destroying the epithelial remnants. Finally, a buccal flap was mobilized and sutured over the bony window. As the complete closure of the wound was not achieved, healing by second intention was allowed, filling the cavity with a sterile gauze impregnated with chlorhexidine digluconate for one week. The sample obtained was sent for histopathological analysis, which showed adaptive changes in the cystic epithelium, hyperplastic in some areas, mostly non-keratinized, with loss of the basal palisade, as well as the presence of a chronic inflammatory infiltrate in the capsular connective tissue (Fig. 2C,D). Periodic check-ups to observe the filling of the cavity and the complete bone regeneration at 6 months were made (Fig. 3C). The last control visit at 2 years showed no signs of recurrence.

## Discussion

Although OKC is a benign lesion, its biological characteristics make this cyst to be considered as an “aggressive” entity (11). This lesion tends to recur and can reach large dimensions before being detected (8,10); therefore, it is advisable to make a correct treatment with the complete removal of the cystic lesion to reduce or eliminate the possibility of recurrence (4,11).

Enucleation has been the most used treatment modality for decades; however, applying this technique without adjuvants contributes to high recurrence rates (11,13). In a study by Zecha *et al.* (8) on 58 OKC treated by enucleation, 20.7% of cases relapsed after a mean of 46 months of follow-up. Previously, another similar study by Jensen *et al.* (13) showed that 33% of OKC treated by simple enucleation recurred in a period between 17 and 58 months. One of the main reasons that would explain these high recurrence rates could be the incomplete removal of the cyst, due to the well-known difficulties for enucleating and perform the complete removal of the cyst, due to the thinness and friability of its capsule (8,9,13). Other reasons that would explain the recurrences could be the presence of daughter cystic into the adjacent bone or mucosa, or the presence of epithelial islands within the lining mucosa (6,8,9). Although the resective therapeutic modality obtains the lowest recurrence rates (5), this approach constitutes a severe mutilation (10,11). Faced with these two opposite therapeutic approaches, in this clinical case we tried to identify an intermediate treatment that would allow for an accepTable long-term success rate and a relatively low morbidity throughout the implementation of a close clinical control as well as careful radiographic evaluation.

In recent years, the decompression or marsupialization of large odontogenic cysts have been widely used as conservative treatments (3,7,16). These techniques are intended to create a window in the cyst wall, exposing its content and maintaining its continuity with the oral cavity. The only portion of the cystic capsule that is removed is the one that is eliminated to create the window, leaving the remaining membrane. The difference between them is that, in decompression, a smaller bone window is practiced, mantaining opened by a surgical drain sutured at its edges, which is shortened as the size of the lesion decreases (3). On the contrary, marsupialization needs a larger window that is kept opened by means of surgical gauze (18). In our opinion, marsupialization allows an easier cleaning of the cystic cavity and avoids some complications that may occur with decompression, such as the loss of drainage due to the decrease in tensile strength of the suture (19).

These two techniques produce changes in the histological structure of the cyst, thickening its capsule thanks to the fibrosis that induces inflammation secondary to oral exposure, which facilitates a possible subsequent removal (3). Since the capsule of OKC is extremely thin and friable, these changes will be advantageous. In addition, by decreasing its osmotic pressure together with the appearance of inflammation, the formation of interleukin-1α, responsible for bone resorption and the expansion of the lesion, is inhibited (16). With all this, it is possible to reduce the size of the lesion, enabling bone growth from its periphery and decreasing the extent of posterior enucleation (6,7,17,18).

It is interesting to note that recurrence rates do not decrease despite the histopathological changes that take place (7). In fact, Nakamura *et al.* (17) showed high frequency in satellite cysts and epithelial remnants even after marsupialization, ranging from 21.4% to 43.5%. In the study of Awni *et al.* (3), similar results were found. These findings indicate that cell proliferative activity persists during and after marsupialization in pre-existing epithelial islands and daughter cysts. In addition, as the time from the onset of marsupialization increases, p53 expression and inflammatory changes increase, indicating a greater increase in recurrence rates (3). In line with these findings, the retrospective study by Zecha *et al.* (8) noted that, after an average follow-up of 49.5 months, 20.7% of the OKC treated by simple enucleation and 40% of those treated by marsupialization recurred. The meta-analysis performed by Al-Moraissi *et al.* (11) also indicated an increase in recurrences with marsupialization (32.3%) compared to enucleation (23.1%). Therefore, we consider that OKC cannot be treated only with a decompression/marsupialization, but it is advisable to perform the complete enucleation of the residual lesion after reaching the objectives of the initial marsupialization, as we have proceeded in our case.

The removal of the lining mucosa in contact with the cystic lesion has been recommended in order to reduce recurrence rates due to the permanence of cystic remnants or epithelial islands (9). In addition to the above mentioned, we consider essential to implement a curettage of the bony bed to eliminate 1 to 2 mm of bone around the residual cystic cavity to diminish the risk of recurrence (4,5,11,13). This behavior can be mechanical (2), chemical (4,15) or by cryosurgery (14). The most conservative variant is the mechanical, which is performed with a rotary bur practicing a peripheral ostectomy of the surrounding tissue (2). This treatment, in addition to enucleation, provides better results than simple enucleation (11). Another option is cryosurgery, applying liquid nitrogen to the bone at -20°C. Which is associated to lower recurrence rates than those of simple enucleation, as shown by Schmidt and Pogrel (14) in 26 cases treated by enucleation and cryotherapy, with only 3 recurrences (11.5%). The depth of bone penetration and cell death caused by liquid nitrogen is close to 0.82 mm and the advantage of its use is that it maintains the bone architecture and facilitates its regeneration (14). On the other hand, some disadvantages are the poor availability of this equipment and the facility to produce nerve damage or soft tissue necrosis if it is not correctly applied (14,20). Finally, and as we use in our case, a chemical curettage can be performed using Carnoy’s solution (15). This fixing liquid is a caustic mixture of ferric chloride in alcohol, chloroform and concentrated acetic acid, which fixes and denatures the tissues to a depth of approximately 1.54 mm, enough to destroy the necessary bone wall (9,15). However, unlike liquid nitrogen, it does not favor bone regeneration and the inclusion of chloroform in its formula represents a great disadvantage since this product has been classified as carcinogenic (20). Despite this, with the use of Carnoy’s solution, recurrence rates of less than 10% have been published (5,10,15).

The combination of the cystectomy, performed after a previous decompression/marsupialization, with adjuvant treatments in the management of OKC entails an energetic approach for these lesions. In our opinion, the combination of these complementary maneuvers to enucleation might minimize the risk of recurrence.

## Conclusions

This case report reviews the current therapeutic options for OKC and shows the treatment of a large OKC in a pediatric patient by an energetic approach with a conservative perspective. Marsupialization followed by surgical enucleation with mucosa excision and Carnoy’s solution can help manage treatment of OKC, lesions characterized by an aggressive behavior. There was no evidence of recurrence after 2 years of follow-up. It is mandatory to perform clinical and radiographic monitoring for years after surgery, since recurrences can appear after several years later.
